# Time, space and feature similarity determine attractive and repulsive serial biases in trustworthiness impressions

**DOI:** 10.1186/s12915-026-02650-3

**Published:** 2026-06-10

**Authors:** Fiammetta Marini, Clare A. M. Sutherland, Linda Jeffery, Mauro Manassi

**Affiliations:** 1 School of Psychology, University of Aberdeen, King’s College, Aberdeen, UK; 2 School of Psychological Science, University of Western Australia, Perth, Australia; 3 School of Population Health, Curtin University, Bentley, WA Australia

**Keywords:** Trustworthiness impressions, Serial effects, Negative aftereffects, Serial dependence

## Abstract

**Background:**

Facial trustworthiness impressions influence important social decisions, including economic choices, hiring decisions and criminal justice outcomes. Most research has focused on the face attributes associated with various impressions, implicitly regarding these impressions as stable over time. However, our perception is dynamically shaped by past visual experience through two opposite serial effects: negative aftereffects (bias away from the past) and serial dependence (bias towards the past). Here, we investigated how past visual experience biases first impressions of trustworthiness by using a trustworthiness rating task. Specifically, we tested how basic stimulus properties such as exposure duration to a previous face, feature similarity, and spatial proximity to previous faces determine the direction and strength of the biases.

**Results:**

We found that (1) exposure duration determined the direction of the bias with negative aftereffects following prolonged adaptor exposure and (positive) serial dependence after brief adaptor exposure. Furthermore, (2) increased identity similarity between previous and current faces enhanced attractive biases, whereas different identities led to repulsion. Finally, (3) relative spatial distance between adaptor and test faces modulated the strength of serial dependence but not negative aftereffects.

**Conclusions:**

Our paradigm enabled us to provide evidence for, and to dissociate, attractive and repulsive serial biases in trustworthiness impressions. Our findings demonstrate the specific conditions under which these impressions can be dynamically shaped by past experience. Importantly, the influence of basic stimulus properties of time, space, and feature similarity on serial effects indicates that these biases cannot be entirely explained by high-level factors such as decision or memory.

**Supplementary Information:**

The online version contains supplementary material available at 10.1186/s12915-026-02650-3.

## Background

Deciding whether we can trust a stranger is central in our social interactions. However, we often base such important trustworthiness judgements on superficial cues, such as facial appearance [1–4]. Facial impressions of trustworthiness are formed in as little as 33 ms [3, 5], and people tend to agree on how trustworthy a face appears [6, 7]. Critically, however, these impressions do not reliably reflect others’ actual trustworthiness [6, 8]. Nevertheless, they influence our everyday social decisions, influencing economic choices [9], employment outcomes [10–12], romantic relationships [13–16] and criminal justice sentences [17, 18].

Trustworthiness impressions research has traditionally focused on identifying the facial morphological features that influence trustworthiness appearance and the real-life consequences of impressions [4, 8]. For example, previous work showed that facial morphological characteristics that resemble emotional expressions influence trustworthiness impressions [19–21]. Neutral faces with features associated with positive emotions, such as high surprised-looking eyebrows and an upward-shaped mouth, are perceived as trustworthy. In contrast, neutral faces with features resembling negative emotions, such as anger-like eyebrows, are generally judged as untrustworthy [3, 8, 17]. This research, by focusing on the face characteristics that influence impressions, has rarely considered how perception of even stable morphological characteristics may vary across time and alter the impressions formed. However, visual perception is constantly affected by past visual experience, both in a repulsive way through negative aftereffects [22–27] and in an attractive manner through serial dependence [28–32].

Negative aftereffects occur when prolonged exposure to a stimulus generates a bias in the perception of subsequent stimuli’s characteristics *away from* recent visual experience [22, 23, 25, 30]. In face perception, negative aftereffects have been observed for facial identity [33–35], emotional expression [22, 25, 36–39], gender and race [40], and facial trait impressions, including attractiveness [41] and trustworthiness [42–44]. For example, prolonged exposure to a trustworthy-looking face can cause the subsequent face to be perceived as less trustworthy-looking than prior to exposure [43, 44]. It has been proposed that this phenomenon occurs because our visual system “recalibrates” to adapt to the prevailing inputs in the visual environment, enhancing sensitivity and discrimination of frequently encountered stimuli [25, 27, 45]. Negative aftereffects usually occur when two successive stimuli are very dissimilar. In such cases, the visual system interprets large differences between previous and present features as coming from distinct objects, and it differentiates the current input from the past experience [46–48]. Previous work has explained negative aftereffects in terms of neuronal fatigue [49, 50], suggesting that adaptation reduces the responses of those channels most sensitive to the adapter stimulus in a population of narrowly tuned, overlapping channels, or efficient neuronal coding in response to redundant sensory information [51, 52], among others.

Serial dependence, by contrast, is a phenomenon in which actions, perception, decisions, and memory of features or objects are systematically biased *towards* the features presented briefly in the recent past [28–32, 53]. Serial dependence has been shown to play a role in shaping face perception, occurring for facial identity [54–57], emotional expressions [58, 59], age [60], and attractiveness [61–65]. It has been proposed that serial dependence is an efficient, purposeful mechanism that helps stabilise our vision, by merging past and present visual information, thus reducing noise in the visual input [29, 30, 60, 66]. Serial dependence manifests especially when two successive stimuli are briefly presented, share similar features [54, 67] and appear in spatial proximity [29–31]. In fact, since small differences in past and present visual information are more likely interpreted as noise, the visual system merges these inputs to stabilise vision, resulting in an attractive bias towards the previous stimulus. Serial dependence was proposed to operate broadly in perception and cognition, with preliminary work showing serial dependence manifesting on perception [29–31, 59, 60, 66, 68, 69], decision [32, 70–74] and visual working memory [75–78]. Surprisingly, the only study which has investigated serial dependence in first impressions of trustworthiness found no evidence for it [65], despite evidence of serial dependence for attributes like identity and emotional expression, which are known to influence trustworthiness impressions. However, this study did not use face stimuli selected to vary primarily in trustworthiness so may have lacked sensitivity to serial effects.

These opposite serial biases have traditionally been investigated separately. Previous studies have mainly focused only on one type of serial effect at the time, employing different experimental designs, stimuli and analyses. As a result, it is difficult to integrate findings across studies and draw a general conclusion about the factors that determine the strength and direction (attractive or repulsive) of these serial effects and their underlying mechanisms in facial trait impressions. Importantly, while previous work has shown that different facial attributes (e.g. gender and expression) can exhibit different directions of serial bias [79], it remains unclear whether a single facial attribute can be subject to both attractive and repulsive effects within the same experimental paradigm. Specifically, no study so far has systematically explored how basic stimulus properties determine these two opposing serial biases in face perception. In this regard, prior research has suggested that basic stimulus properties like stimulus duration [26, 29, 45], in addition to visual similarity [46, 66] and spatial proximity of sequential stimuli [29–31], influences the direction of serial biases in simpler visual stimuli. However, given that faces are higher-order visual stimuli with social significance, it is particularly important to investigate whether the same principles that govern serial biases for basic visual features such as orientation perception also apply to facial trait impressions. Moreover, investigating whether previously seen faces influence current trustworthiness impressions and the factors involved in determining the serial biases is crucial, as such effects could distort social evaluations in everyday life.

The aims of this study were to (1) determine if serial dependence operates for facial first impressions and (2) characterise for the first time the conditions under which repulsive (negative aftereffects) or attractive (serial dependence) biases arise for high-level social judgements, within a single experimental paradigm. We focused our investigation on trustworthiness impressions given their central role in face evaluation and their significant influence on everyday social interactions [1–4, 8, 80]. Specifically, we investigated whether and how basic properties of the previously seen faces which previous research suggests influence serial dependence and aftereffects—time, space, and feature similarity—affect serial effects in trustworthiness impressions. To this purpose, we employed a rating task in which participants rated the trustworthiness of an adaptor face followed by a test face, both varying in trustworthiness levels. First, we tested whether increasing adaptor duration can lead to a dissociation between attractive and repulsive serial effects (Experiment 1). We manipulated the exposure duration of the previous adaptor faces (brief presentation of 1 s versus long presentation of 9 s), building on evidence that brief exposure duration tends to elicit serial dependence [54, 55, 64, 67, 69], whereas longer exposures typically produce negative aftereffects [34, 41, 43, 48, 81, 82]. Next, we tested whether two additional factors modulating serial dependence—feature similarity [54, 59, 66, 83] and spatial proximity [30, 31, 54]—between the adaptor and test face influence the strength and direction of these serial effects (Experiment 2).

We hypothesised that brief adaptor duration (1 s) would elicit serial dependence, whereas long adaptor duration (9 s) would generate negative aftereffects. Additionally, we predicted that feature similarity between stimuli would enhance an attractive bias, since prior work has shown that feature similarity increases serial dependence [54, 59, 66, 83], while dissimilarity would lead to a repulsive bias [34, 39, 41, 48, 81, 82]. Finally, we proposed that spatial proximity between stimuli would affect serial dependence, predicting stronger serial dependence when adaptor and test faces appeared closer in space, consistent with evidence that serial dependence is modulated by spatial tuning [29–31]. Importantly, if the basic physical properties of the previous adaptor face influence the following serial effects, then a bias occurring on a mere decisional or memory level would not be able to fully account for the direction (repulsive/attractive) and strength modulation of serial effects. Conversely, if serial effects are determined solely by decisional and memory components, variations in the perceptual properties of the previous adaptor face related to time exposure, feature similarity, and spatial proximity should not influence the serial effects in trustworthiness judgements.

## Results

### Experiment 1A and 1B

**Fig. 1:**
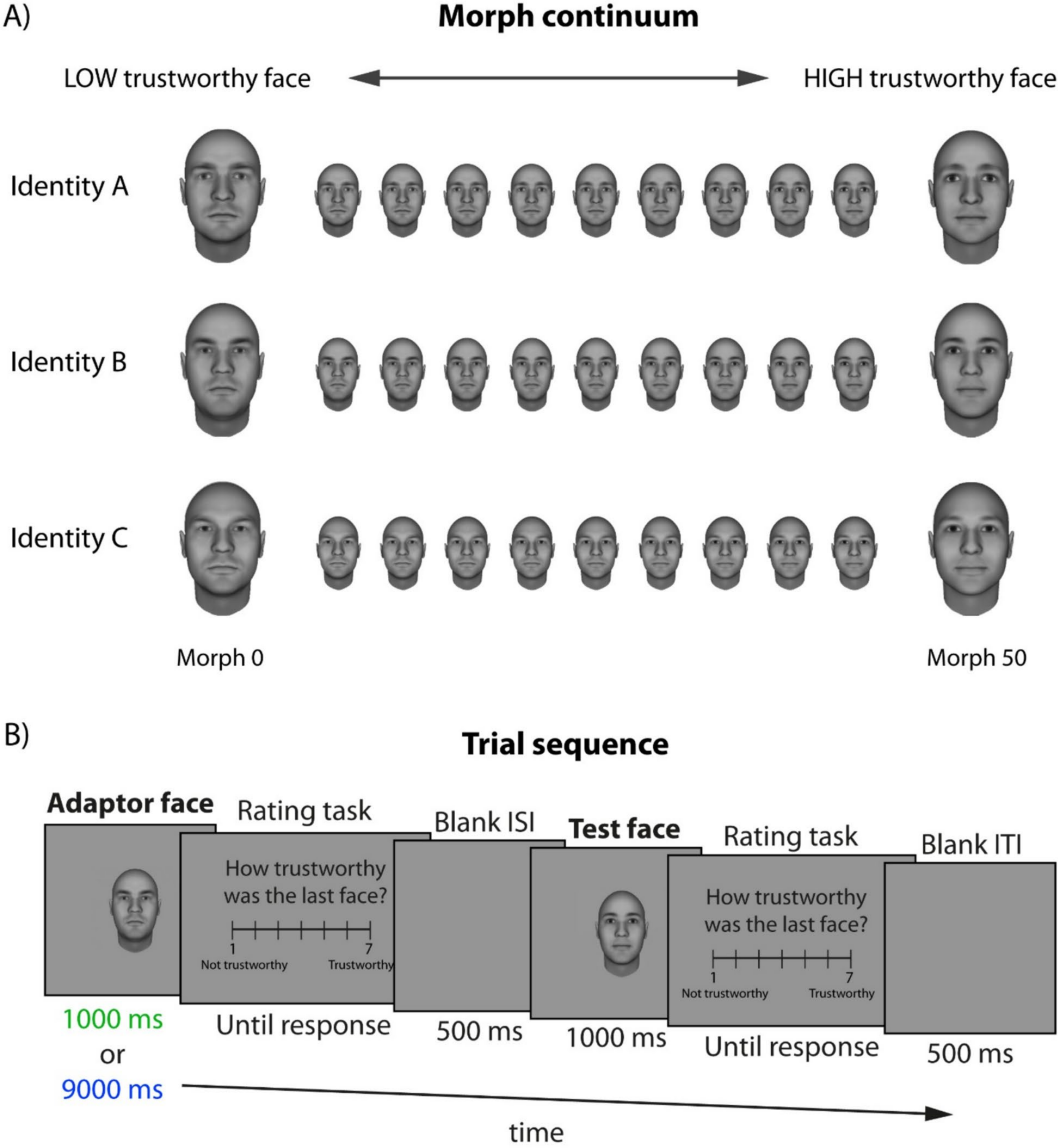
Stimuli and trial sequence in Experiment 1A and 1B. **A** Stimuli used in Experiment 1A and 1B. **B** Trial sequence in Experiment 1A and 1B. On each trial, observers saw an adaptor face for 1 s (Experiment 1A) or 9 s (Experiment 1B) and were asked to indicate its level of trustworthiness on a rating from 1 to 7. Subsequently, after an ISI of 500 ms, a test face was displayed for 1 s, and participants were asked to rate its trustworthiness on the same scale. Finally, after an ITI of 500 ms, the following trial began

In Experiment 1A we tested whether serial dependence could be shown for facial trustworthiness, whereas in Experiment 1B we replicated trustworthiness aftereffects found in previous research [43, 44]. We aimed to test whether exposure duration to a previous face is a key factor in determining the direction of serial effects in trustworthiness impressions. On each trial, we displayed adaptor and test faces with varying levels of trustworthiness randomly selected from three computer-generated identity continua composed of 51 morphs ranging from very low to very high in trustworthiness appearance (Fig. 1A; see “General methods” section). The adaptor face was displayed for 1 s in Experiment 1A and for 9 s in Experiment 1B. Participants then rated the trustworthiness of the subsequent 1 s test face. We measured how participants’ judgements of trustworthiness of the current test face were biased towards (i.e. serial dependence) or away from (i.e. negative aftereffects) the adaptor face’s level of trustworthiness.

**Fig. 2:**
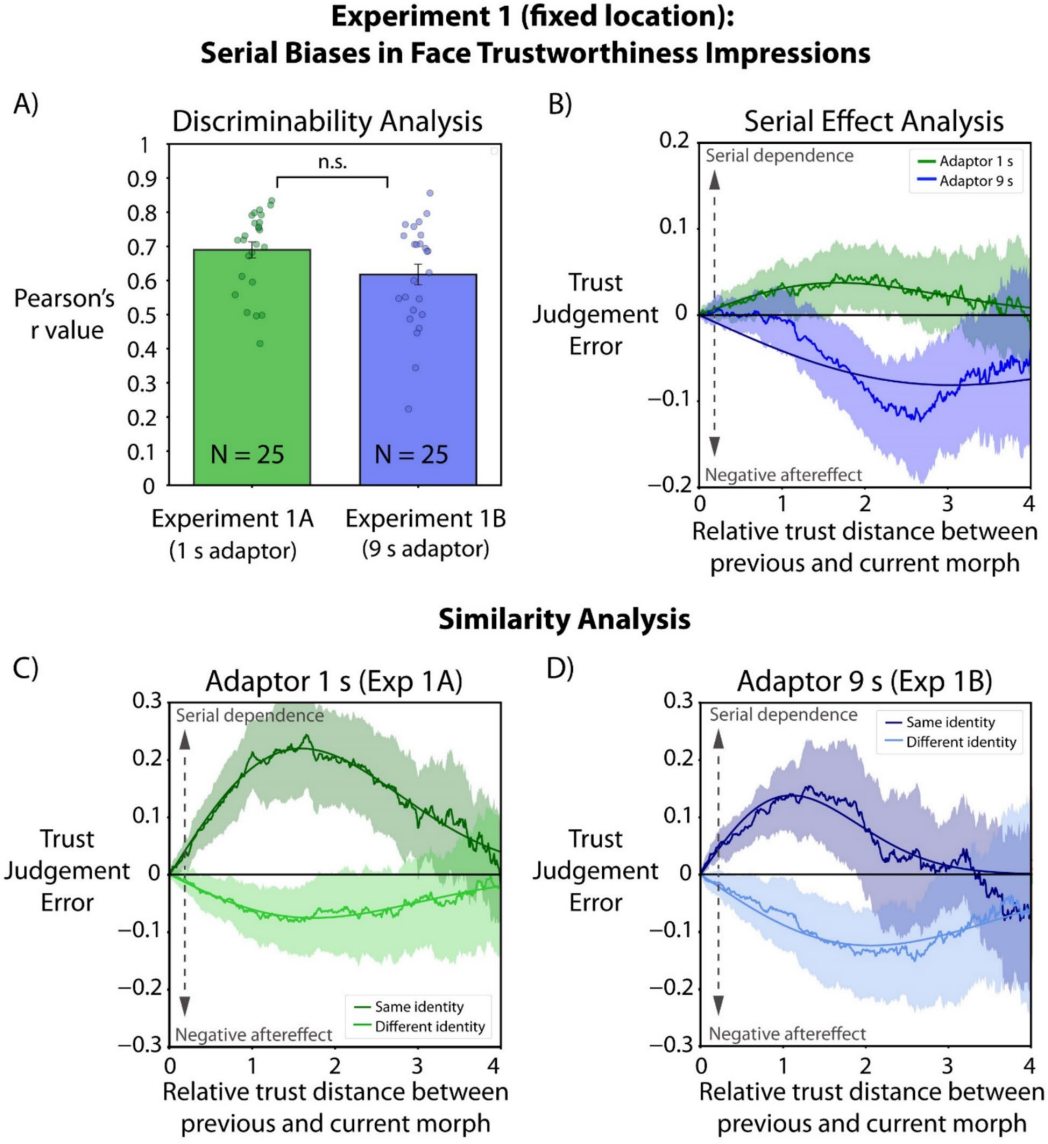
Results of Experiment 1A and 1B. **A** Discriminability analysis. Individual participant correlations (dots) between trustworthiness ratings (1–7) and morph values (0–50 morph units) are plotted. Bars show the average Pearson’s *r* for each group, with error bars representing the standard error of the mean. **B** Serial effect analysis. This graph represents the average of the bootstrapped distribution of flipped running means and DoG fits for both Experiment 1A (green curve) and Experiment 1B (blue curve). The *x*-axis represents trustworthiness judgement errors (rating − estimated target score), and the *y*-axis shows the relative trustworthiness distance in estimated scores between previous and current morph (rated trustworthiness on current trial − rated trustworthiness on previous trial). Shaded areas show the 95% confidence intervals of the bootstrapped distribution of flipped running means. Experiment 1A (1 s adaptor) shows a positive DoG half-amplitude, indicating serial dependence. Experiment 1B (9 s adaptor) shows a negative half-amplitude, indicating a negative aftereffect. **C** Similarity analysis for Experiment 1A. The participant’s pooled data and DoG fits are shown for the same identity condition (dark green) and different identity condition (light green). **D** Similarity analysis for Experiment 1B. The participants’ pooled data and DoG fits are shown for the same identity condition (dark blue) and different identity condition (light blue)

First, to assess whether observers were able to discriminate the level of trustworthiness of the face morphs from the continuum, we correlated each participants’ ratings across the experiment with the morphs shown on each trial (see “Discriminability analysis”, Fig. 2A). This analysis was conducted to confirm that observers were sensitive to subtle variations in the trustworthiness of our stimuli, and that this sensitivity was comparable across Experiments 1A and 1B, allowing for meaningful comparisons between results. Participants in Experiment 1A had a mean correlation value of 0.7 (std −/+ 0.11; *t*(24) = 29.49, *p*-value < 0.001, *n* = 25), whereas participants in Experiment 1B had a mean correlation value of 0.6 (std −/+ 0.15; *t*(24) = 20.03, *p*-value < 0.001, *n* = 25), suggesting that they could discriminate the morph levels successfully. Additionally, participants in the two groups did not differ in their discrimination sensitivity (Experiment 1A vs 1B; *t*(24) = 1.88, *p*-value = 0.07).

Next, to examine whether observers showed serial effects in trustworthiness judgements, we pooled all participants’ judgement errors and the differences in trustworthiness between the adaptor and test face stimuli. Then, we computed the running mean of the pooled trustworthiness judgement errors across all previous and current morph trustworthiness distances. Due to its symmetrical nature around zero and to reduce noise, we simplified the data by flipping the left side of the running mean (negative morph distances) and merging it with the right side (positive morph distance), creating a "flipped running mean" made of only positive morph distances [68, 84]. We then fitted a Derivative-of-Gaussian (DoG) function to the flipped running mean trustworthiness judgement errors as a function of relative trustworthiness distance between adaptor and test face. The half-amplitude (a parameter) of the DoG indicated the strength and direction of the serial effect. A positive half-amplitude value shows an attractive bias (serial dependence) in the judgements of the current face towards the level of trustworthiness observed in the previous face, whereas a half-amplitude with negative value indexes a repulsive bias (negative aftereffect) away from the previous level of trustworthiness seen (Fig. 2B). A half-amplitude value of zero indicates no serial bias (see “Serial effect analysis”).

In Experiment 1A (brief exposure), we found a positive half-amplitude of 0.04 (bootstrap against zero; *p*-value = 0.04), indicating a bias in trustworthiness judgement on the test face towards the adaptor face (i.e. serial dependence; Fig. 2B) contrary to the previous work findings [65]. In Experiment 1B (longer exposure), we found a negative half-amplitude of − 0.08 (bootstrap against zero; *p*-value < 0.01) indicating a bias in trustworthiness judgements on the test face away from the adaptor face (i.e. negative aftereffects; Fig. 2B). To compare these effects, we subtracted the Experiment 1B half-amplitude distributions from those of Experiment 1A, and we calculated the one-tailed *p*-value as the proportion of difference distribution values greater than zero. This analysis showed a significant difference between the two experiments (between-subject Bootstrap; *p*-value = 0.02). Overall, our results showed that brief exposure to the adaptor face (1 s) led to serial dependence, whereas longer exposure (9 s) led to negative aftereffects in trustworthiness impressions.

We then tested whether serial effect strength and direction were modulated by identity similarity between adaptor and test face. Since adaptor and test faces were randomly drawn from three identity morph continua, we split the trials into two conditions: “same identity” trials (adaptor and test face from the same identity morph continuum) and “different identity” trials (adaptor and test face from different identity morph continuum). For both conditions, we calculated the serial effect’s strength and direction as reported before. In Experiment 1A (Fig. 2C), in the same identity condition, we found a positive half-amplitude of 0.22 (bootstrap against zero; *p*-value < 0.001), indicating a positive bias towards the past (serial dependence; Fig. 2C). In the different identity condition, we found a negative half-amplitude of − 0.08 (bootstrap against zero; *p*-value < 0.01), indicating negative aftereffects. Given the different number of trials between identity conditions (see “Similarity analysis” in the “General methods” section), we ran an additional bootstrap for the different identity condition. Specifically, we resampled trials to match the same identity condition’s trial count for each selected participant, therefore allowing for a fair comparison between the two similarity conditions. This additional analysis confirmed a negative half-amplitude of − 0.09 (bootstrap against zero; *p*-value < 0.01), indicating negative aftereffects. By comparing the half-amplitudes bootstrapped distributions of the same identity and different identity condition in Experiment 1A, we found a significant difference (within-subject bootstrap; *p*-value < 0.001). These results suggest that identity similarity between adaptor and test face, together with adaptor duration, influences the resulting serial effect direction. Specifically, serial dependence occurred when adaptor and test faces shared identity, while different identities led to negative aftereffects.

In Experiment 1B (Fig. 2D), in the same identity condition we found a positive half-amplitude of 0.13, which was not significantly different from zero (bootstrap against zero; *p*-value = 0.06). On the other hand, in the different identity condition, a negative half-amplitude of − 0.13 (bootstrap against zero; *p*-value < 0.001) was found, even after trial resampling (bootstrap against zero; *p*-value < 0.001), indicating negative aftereffects. The half-amplitudes bootstrapped distributions for the same and different identity condition were significantly different (within-subject bootstrap; *p*-value = 0.03). Therefore, same identities between adaptor and test faces led to an attractive bias, whereas different identities resulted in negative aftereffects. Moreover, we compared the influence of identity similarity across Experiments 1A and 1B. No statistically significant difference was found between Experiment 1A and 1B’s half-amplitude distributions for either the same identity condition (between-subject bootstrap; *p*-value = 0.08) or the different identity condition (between-subject bootstrap; *p*-value = 0.18). While the distributions suggest a shift in amplitudes, it is likely that this was not sufficiently strong to be observed in these experiments.

Taken together, these results indicate that serial effects in trustworthiness impressions are influenced by both the exposure duration to the adaptor face and the similarity in identity between the adaptor and test faces. Specifically, brief exposure (1 s, Experiment 1A) led to serial dependence, biasing trustworthiness judgements toward the adaptor face level of trustworthiness. Conversely, longer exposure (9 s, Experiment 1B) resulted in negative aftereffects, where judgements are biased away from the adaptor face’s trustworthiness. Moreover, when the adaptor and test face shared the same identity, serial dependence was found. When the adaptor and test face had different identities, a repulsive bias occurred. Importantly, these results suggest that the present serial biases cannot readily be explained by mere motor response biases or decision/memory biases. In fact, despite participants consistently making perceptual judgements about the trustworthiness on the current face, the serial effects varied depending on (1) adaptor duration and (2) identity similarity.

## Experiment 2A and 2B

In Experiment 2A and 2B, we tested whether the direction and magnitude of serial effects depend on the relative spatial location of adaptor and test faces. On each trial, we randomised on the spatial location of the adaptor and test faces while maintaining the same adaptor and test faces duration of Experiment 1A and 1B. Participants were instructed to fixate the faces, and thus the distance in spatial location was in world-centred coordinates, therefore, relative to the position of faces in the world, not by their retinal position.

**Fig. 3:**
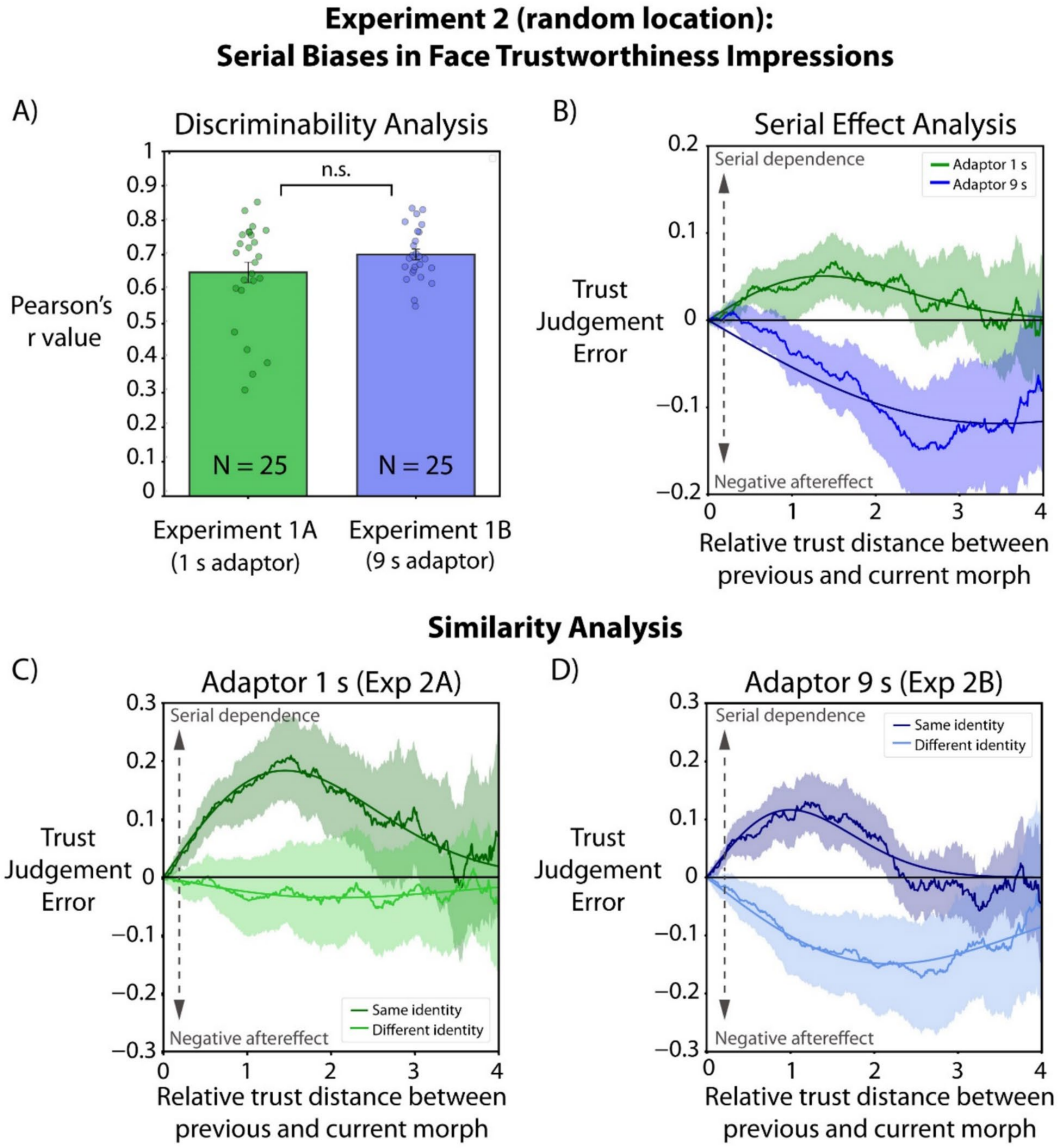
Results of Experiment 2A and 2B. **A** Discriminability analysis. This graph shows individual participant correlations (dots) between trustworthiness ratings (1–7) and morph values (0–50 morph units). Bars represent the average Pearson’s *r* for each group, with error bars indicating the standard error of the mean. **B** Serial effect analysis. The average of the bootstrapped distribution of flipped running means and DoG fits for both Experiment 2A (green curve) and Experiment 2B (red curve) is plotted. The *x*-axis represents the trustworthiness judgement error for each trial (trustworthiness rating − estimated score of target face), while the *y*-axis shows the relative trustworthiness distance in estimated scores between previous and current morph (rated trustworthiness on current trial − rated trustworthiness on previous trial). Shaded areas represent 95% confidence intervals of the bootstrapped running means. Experiment 2A (1 s adaptor) displays a positive DoG half-amplitude, indicating serial dependence. Experiment 2B (9 s adaptor) exhibits a negative half-amplitude, indicating a negative aftereffect. **C** Similarity analysis for Experiment 2A. The participants’ pooled data and DoG fits are shown for the same identity condition (dark green) and different identity condition (light green). **D** Similarity analysis for Experiment 2B. The participants’ pooled data and DoG fits are shown for the same identity condition (dark blue) and different identity condition (light blue)

First, by correlating participants’ ratings with the morphs showed on each trial, we confirmed that participants were able to discriminate the level of trustworthiness of the face morphs from the continuum both in Experiment 2A (mean correlation value = 0.6 −/+ 0.15; *n* = 25; *t*(24) = 21.61, *p*-value < 0.001) and Experiment 2B (mean correlation value = 0.7 −/+ 0.07; *n* = 25; *t*(24) = 45.47, *p*-value < 0.001). Discrimination sensitivity did not significantly differ between the two groups (*t*(24) = − 1.53, *p*-value = 0.13; Fig. 3A).

Second, we tested whether observers exhibited a serial effect and the direction of the bias in both Experiment 2A and 2B, using the same analysis as in Experiment 1. In Experiment 2A (brief exposure), as predicted, we found a positive half-amplitude of 0.05 (bootstrap against zero; *p*-value = 0.04), indicating a serial dependence bias (Fig. 3B). In Experiment 2B (longer exposure), as predicted, we found a negative half-amplitude of − 0.1 (bootstrap against zero; *p*-value < 0.001), indicating negative aftereffects (Fig. 3B). The comparison between the two bootstrapped half-amplitude distributions showed a significant difference in serial effect between the two experiments (between-subject bootstrap; *p*-value = 0.02). Overall, consistent with Experiment 1A and 1B, these findings suggest that brief exposure to the adaptor face (1 s) biases trustworthiness impressions of subsequent test faces towards the previously seen level of trustworthiness (i.e. serial dependence), whereas longer exposure (9 s) biases the impressions of trustworthiness of the current face away from previously seen face level of trustworthiness (i.e. negative aftereffects).

To test whether the serial effect strength and direction were modulated by identity similarity between the current test face and the previously seen adaptor face, for each adaptor duration condition (1 s and 9 s), we analyzed the serial effect at a group level for the “same identity” and “different identity” trials. In Experiment 2A (1 s adaptor duration), in the same identity condition, we found a positive half-amplitude of 0.18 (bootstrap against zero; *p*-value = 0.001), indicating a serial dependence bias (Fig. 3C). On the other hand, in the different identity condition, a negative half-amplitude of − 0.03 was found, but this was not significantly different from zero (bootstrap against zero; *p*-value = 0.14) suggesting no significant serial effects. A significant difference was found between the same and different identity conditions (within-subject bootstrap; *p*-value = 0.01). In Experiment 2B (9 s adaptor duration), in the same identity condition, a positive half-amplitude of 0.1 indicated serial dependence, but was not significantly different from zero (bootstrap against zero; *p*-value = 0.09). On the other hand, in the different identity condition, a negative half-amplitude of − 0.14 (bootstrap against zero; *p*-value < 0.001) was found, which remained significant even after resampling trials to match the same identity condition’s trial count (half-amplitude: − 0.14; bootstrap against zero; *p*-value < 0.001), indicating the presence of negative aftereffects. No significant difference was found between the same and different identity conditions in Experiment 2B (within-subject bootstrap; *p*-value = 0.07). We also examined whether identity similarity influenced the serial effect differently across Experiment 2A and 2B. No significant differences were found in either the same identity condition (between-subject bootstrap; *p*-value = 0.14) or the different identity condition (between-subject bootstrap; *p*-value = 0.09). Although the distributions show a potential shift in amplitude, the effect was likely too subtle to be detected in these experiments. Overall, serial dependence occurred when adaptor and test faces shared identity, while different identities led to a repulsive bias. Consistent with Experiment 1, these results demonstrate that both adaptor duration and identity similarity determine the strength and direction of serial effects.

**Fig. 4:**
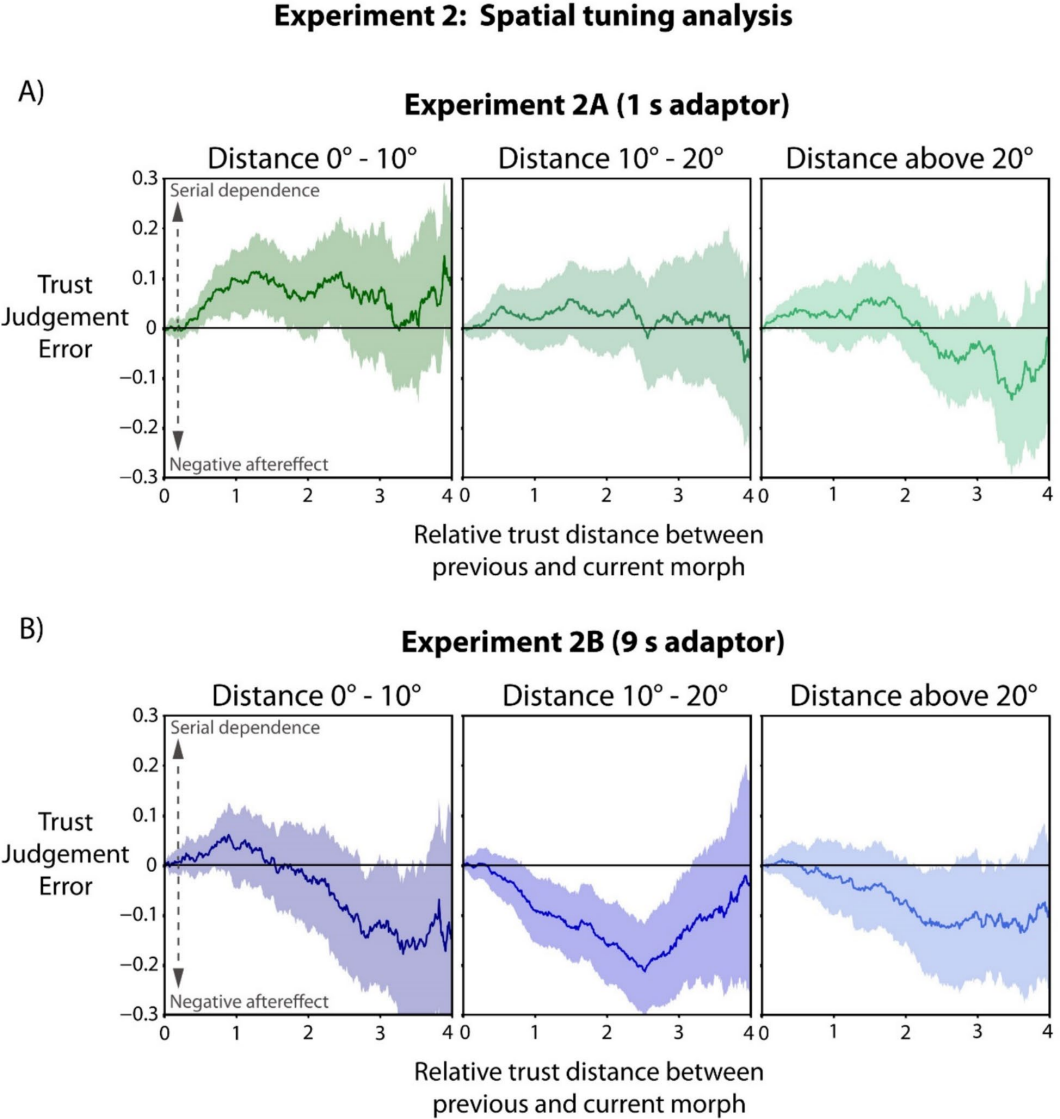
Spatial tuning results of Experiment 2A and 2B. The average of the bootstrapped distribution of flipped running means for both Experiment 2A (**A**) and Experiment 2B (**B**) is plotted, with trials binned in different ranges of distance conditions between adaptor and test face (0–10°/10–20°/beyond 20° distance). The *x*-axis represents the trustworthiness judgement error for each trial (trustworthiness rating − estimated score of target face), while the *y*-axis shows the relative trustworthiness distance in estimated scores between previous and current morph (rated trustworthiness on current trial −rated trustworthiness of previous trial). The shaded area around the running mean is the confidence intervals of the bootstrapped distribution of flipped running means. In Experiment 2A, where the adaptor face was displayed for 1 s, the serial dependence bias decreases with increasing spatial distance between the previous and current face. Conversely, in Experiment 2B, where the adaptor face was displayed for 9 s, negative aftereffect bias does not change with increasing spatial distance between adaptor and test face

Finally, we tested whether the strength of serial effects for trustworthiness impressions is influenced by the spatial proximity between subsequently perceived faces. To this purpose, for both Experiment 2A and 2B, each participant’s trials were divided into three spatial-distance bins: short distance (0–10° spatial distance), medium distance (10–20° spatial distance), and long distance (beyond 20° spatial distance). As this analysis considered less trials (approximately 294 per participant compared to approximately 70 trials per participant), the moving average curves were considerably noisier (see Fig. 2B vs Fig. 4), making the DoG fit unreliable. Therefore, we adopted an alternative method considering the area subtended by the moving average, which is less sensitive to curve irregularities and does not rely on a specific curve shape. As before, for each bootstrap iteration participants were sampled with replacement, and their trials were grouped together by spatial distance (short, medium, long). Instead of fitting a DoG, we calculated the mean area under the flipped moving averages for each of the three spatial distance curves by calculating the average of all *y*-axis values. Positive magnitude values (curve points above zero) indicate an attractive bias, while negative values (curve points below zero) indicate a repulsive bias. This method resulted in three distributions of 1000 “magnitude” values, one for each spatial distance condition.

In Experiment 2A (brief exposure), we found a positive serial dependence effect in the short-distance condition, with a bootstrapped mean area of 0.06 (bootstrap against zero; *p*-value = 0.01). The medium-distance condition showed a smaller non-significant effect (bootstrapped mean area = 0.01, bootstrap against zero; *p*-value = 0.27), and the long-distance condition a slightly negative effect (bootstrapped mean area = − 0.01), which also was not significant (bootstrap against zero; *p*-value = 0.07; Fig. 4A). Given that face location on the screen was randomly extracted on each trial, the number of trials differed across spatial distance conditions, with the short-distance condition having fewer trials. To ensure a fair comparison, we ran an additional matched-sample bootstrap. In each iteration, we sampled participants with replacement, matching the number of trials across all three distance conditions to that of the short-distance condition. By using this matched-sample bootstrap, we found consistent results with the previous analysis: the bootstrapped mean area for the short distance was 0.06 (bootstrap against zero; *p*-value = 0.01), for the medium distance was 0.02 (bootstrap against zero; *p*-value = 0.27), and for the long distance was − 0.01 (bootstrap against zero; *p*-value = 0.07). To test whether the serial dependence effect varied systematically with spatial distance, we fit a slope to the three magnitude values (short, medium, and long) in each of the 1000 bootstrap iterations, generating a distribution of slope values. The average slope was − 1.48, and significantly different from zero (*p*-value = 0.01), indicating a decline in serial dependence strength with increasing spatial distance. Moreover, while the short-distance and long-distance magnitude distributions were significantly different from each other (within-subject bootstrap; *p* = 0.01), differences between short and medium (within-subject bootstrap; *p*-value = 0.16) and medium and long (within-subject bootstrap; *p*-value = 0.24) were not statistically significant. These results suggest that in Experiment 2A serial dependence was present in the short distance position and decreased with increasing spatial distance, suggesting that spatial proximity between the adaptor and test face modulated the strength of the observed serial effect.

In Experiment 2B (longer exposure), we found negative aftereffect across all the spatial distance conditions (Fig. 4B). The bootstrapped mean area was − 0.04 in the short-distance condition (bootstrap against zero; *p*-value = 0.09), − 0.1 in the medium-distance condition (bootstrap against zero; *p*-value < 0.001), and − 0.06 in the long-distance condition (bootstrap against zero; *p*-value < 0.01), indicating robust negative aftereffects. A bootstrap with matched trial numbers confirmed these consistent results: the short-distance bootstrapped mean area was − 0.04 (bootstrap against zero; p-value = 0.11), the medium-distance average was − 0.1 (bootstrap against zero; *p*-value < 0.001), and the long-distance average was − 0.068 (bootstrap against zero; *p*-value = 0.01). To examine whether this negative bias varied with distance, we fitted a slope to the three magnitude values on each bootstrap iteration. The resulting slope distribution had a mean of − 2.66, but it was not significantly different from zero (within-subject bootstrap; *p*-value = 0.35), suggesting no systematic change in effect magnitude across spatial distances. Additionally, comparisons between the magnitude distributions for the three distances (within-subject bootstrap; short vs. medium: *p*-value = 0.06; short vs. long: *p*-value = 0.35; medium vs. long: *p*-value = 0.05) revealed no statistically significant differences.

Taken together, these results suggest that while serial dependence in Experiment 2A decreased with increasing spatial distance, the negative bias found in Experiment 2B was relatively consistent across spatial positions. However, there was not necessarily any reason to expect spatial tuning in negative aftereffects, given that in this experimental condition participants could foveate towards the face stimuli (and thus retinotopic coordinates were kept constant). In fact, previous work showed that face negative aftereffects could have a retinotopic component [85, 86] (but see: [34, 87]). Since participants likely viewed faces in the same retinotopic location, this could have enabled robust negative aftereffects across all spatial conditions. Our findings reveal a distinction in how the spatial distance between successive faces influences serial dependence and negative aftereffects in trustworthiness impressions.

Overall, Experiment 2A and 2B results replicated Experiment 1A and 1B findings on the role of previous face exposure duration and feature similarity between adaptor and test face in determining the strength and direction of the serial effects. Importantly, Experiment 2A further demonstrated that serial dependence in trustworthiness impressions is modulated by spatial proximity between the previous and current face, with stronger effects when successive faces appear in close spatial locations. These findings highlight that spatial proximity is another factor involved in determining the strength of serial dependence in trustworthiness impressions. Overall, these results contribute to understanding the circumstances in which trustworthiness impressions are shaped by previous faces seen in the visual temporal history and much broadly how serial biases operate in high-level facial social judgements.

## Interaction between time, identity similarity and space

In this analysis, we investigated how time, identity similarity and spatial proximity interact in determining the strength and direction of the serial effect in trustworthiness impressions. Given limited prior research, we did not have specific predictions regarding how variation in the basic stimulus properties might interact, and our design was optimised for testing predictions about each property alone. However, our design did allow for some exploratory analyses of how these stimulus properties may interact to influence perception. In Experiments 1A and 1B, we tested whether the strength and direction of the serial effect were modulated by the interaction between adaptor duration (1 s vs 9 s) and identity similarity (same identity vs different identity) between the current test face and the preceding adaptor face. For each participant, trials were categorised based on identity similarity. Since binning the data resulted in a limited number of trials per category for each participant, in line with the spatial tuning analysis approach employed in Experiment 2A and 2B (see also the “General methods” section), for each participant we quantified the strength of serial effects by considering the area under the moving average rather than calculating the half-amplitude of the DoG curve. More specifically, we calculated the mean area under the flipped moving averages by calculating the average of all *y*-axis values. This method resulted in two “magnitude” values for each participant for each experiment, one for each similarity condition.

To investigate the interaction between adaptor duration and identity similarity in determining the strength and direction of serial effect, we ran a 2 (duration: 1 s vs 9 s) × 2 (identity similarity: same vs different) mixed-design ANOVA. This analysis revealed a significant main effect of duration (*F*(1, 48) = 4.93, *p* = 0.031, *η*^2^ = 0.036), confirming serial dependence for 1 s adaptor condition (mean magnitude value = 0.02 −/+ 0.07) and negative aftereffects for the 9 s adaptor condition (mean magnitude value = − 0.04 −/+ 0.1). We also found a robust main effect of identity similarity (*F*(1, 48) = 44.77, *p* < 0.001, *η*^2^ = 0.29), which reflects the shift from attraction to repulsion based on face identity similarity between successive trials in both Experiment 1A (same identity mean magnitude value = 0.15 −/+ 0.15; different identity mean magnitude value = − 0.05 −/+ 0.10) and Experiment 1B (same Identity mean magnitude value = 0.07 −/+ 0.15; different identity mean magnitude value = − 0.09 −/+ 0.11). However, the interaction between duration and identity similarity was not significant (*F*(1, 48) = 0.71, *p* = 0.404, *η*^2^ = 0.005). These findings suggest that while a longer adaptor duration (9 s) shifts the baseline of the serial effect towards repulsion and a brief adaptor duration (1 s) shifts it towards attraction, the change in direction of the serial effect due to identity similarity remains stable across the two adaptor duration conditions.

Next, we replicated these findings in Experiments 2A and 2B using the same analysis. We again ran a 2 (duration: 1 s vs 9 s) × 2 (identity similarity: same vs different) mixed-design ANOVA. The results mirrored those of Experiment 1A and 1B. We found a significant main effect of duration (*F*(1, 48) = 19.28, *p* < 0.001, *η*^2^ = 0.12), with serial dependence occurring in the 1 s adaptor condition (mean magnitude value = 0.02 −/+ 0.06) and negative aftereffects occurring in the 9 s adaptor condition (mean magnitude value = − 0.07 −/+ 0.09). Additionally, we confirmed the robust main effect of identity similarity (*F*(1, 48) = 34.41, *p* < 0.001, *η*^2^ = 0.24), with a change of direction of the effect both in Experiment 2A (same identity mean magnitude value = 0.13 −/+ 0.14; different identity mean magnitude value = − 0.014 −/+ 0.09) and in Experiment 2B (same identity mean magnitude value = 0.03 −/+ 0.11; different identity mean magnitude value = − 0.011 −/+ 0.10). Importantly, the interaction between duration and identity similarity was not significant (*F*(1, 48) = 0.01, *p* = 0.921, *η*^2^ = 0.0006). This result suggests that the modulation of serial dependence due to identity similarity (i.e. shift from attraction to repulsion) is consistent across both brief (1 s) and longer (9 s) adaptor duration conditions.

Finally, we investigated the interaction between adaptor duration, identity similarity, and spatial distance in Experiment 2A and 2B. First, for each participant, trials were categorised based on spatial distance condition (short vs medium vs long) and identity similarity (same identity vs different identity), resulting in six magnitude values for each participant. Next, we ran a 2 (duration: 1 s vs 9 s) × 2 (identity similarity: same vs different) × 3 (spatial distance: short vs medium vs long) mixed-design ANOVA. This analysis confirmed the significant main effects of duration (*F*(1, 48) = 14.38, *p* < 0.001, *η*^2^ = 0.056) and Identity Similarity (*F*(1, 48) = 22.11, *p* < 0.001, *η*^2^ = 0.077) previously observed. We also found a significant main effect of spatial distance (*F*(2, 96) = 4.46, *p* = 0.014, *η*^2^ = 0.021). Interestingly, we found a significant interaction between identity similarity and spatial distance factors (*F*(2, 96) = 5.98, *p* = 0.004, *η*^2^ = 0.028). This result might be because the attractive bias for “same identity” trials decayed more with spatial distance between successive trials compared to the repulsive bias for “different identity” trials. Specifically, in Experiment 2A (1 s adaptor), the bias for the same identity decreased across distances (short: 0.18 −/+ 0.25; medium: 0.13 −/+ 0.20; long: 0.05 −/+ 0.21), while the bias for the different identity remained relatively stable and near zero (short: 0.02 −/+ 0.19; medium: − 0.03 −/+ 0.14; long: − 0.01 −/+ 0.20). A similar pattern was present in Experiment 2B (9 s adaptor), where the same identity bias shifted from attraction to repulsion as distance increased (short: 0.13 −/+ 0.23; medium: − 0.03 −/+ 0.20; long: − 0.06 −/+ 0.25), whereas the different identity bias showed a more consistent repulsive effect across all spatial bins (short: − 0.13 −/+ 0.21; medium: − 0.15 −/+ 0.16; long: − 0.05 −/+ 0.17). To further investigate the interaction between identity similarity across spatial distance conditions, we ran pairwise *t*-tests. A Bonferroni correction was applied for multiple comparisons (adjusted α = 0.0167). In Experiment 2A, the effect of identity similarity was significant for short distances (*t*(24) = 4.31, *p*-value < 0.001) and medium distances (*t*(24) = 3.16, *p*-value = 0.01) but did not reach significance for long distances (*t*(24) = 2.56, *p*-value = 0.051). Similarly, in Experiment 2B, the effect of identity similarity was significant for short distances (*t*(24) = 3.41, *p*-value < 0.01) and medium distances (*t*(24) = 3.52, *p*-value < 0.01), but it was not significant for long distances (*t*(24) = 1.56, *p*-value = 0.39). These results suggest that while identity similarity is an important factor that drives the serial effect, its influence diminishes as spatial distance between successive faces increases. Additionally, there was no significant interaction between duration and spatial distance factors (*F*(2, 96) = 0.65, *p* = 0.522, *η*^2^ = 0.003). Finally, the interaction between duration, identity similarity, and spatial distance was not significant (*F*(2, 96) = 1.44, *p* = 0.241, *η*^2^ = 0.007). These results suggest that the influence of identity similarity and spatial distance between successive trials on the serial effect remains consistent across the two adaptor duration conditions. Overall, these findings indicate that while exposure duration shifts the general baseline of the bias between attraction and repulsion, the influence of identity similarity diminishes as the spatial distance between successive faces increases.

## Discussion

The present study investigated the factors involved in determining the strength and direction of serial effects in trustworthiness impressions. (1) In Experiment 1A and 1B we found that prolonged exposure to the preceding face biased the trustworthiness impressions of the subsequent face in a repulsive manner (i.e. negative aftereffects), whereas brief exposure led to an attractive bias (i.e. serial dependence). (2) The similarity in identity between the previous and current face played a role in determining the direction of the serial effects. Serial dependence occurred when the faces were similar in identity, whereas negative aftereffects emerged when the faces differed in identity-related features. (3) In Experiment 2, we tested whether the relative spatial distance (in the same retinotopic coordinates) between previous and current face influenced the strength of serial effects. By varying the screen location of the adaptor and test face, we found that serial dependence (but not negative aftereffect) was stronger when the faces appeared in close spatial proximity compared to when they appeared in more distant locations. This result suggests that the strength of serial dependence can be modulated by the spatial distance between successive stimuli. We found no modulation in the strength of negative aftereffects with increasing the relative spatial distance. Overall, the present findings shed light into the mechanisms that determine attractive or repulsive biases in trustworthiness impressions, by indicating that multiple factors related to the previous face—exposure duration, feature similarity, and spatial proximity—interact in determining the strength and direction of serial effects for trustworthiness impressions. Importantly, this is the first study in the domain of face perception to systematically examine how multiple visual factors jointly influence the perception of a single facial attribute to produce repulsive or attractive serial effects.

## The factors influencing serial effects in trustworthiness impressions

In the present study we found that exposure duration, feature similarity, and spatial proximity are factors involved in determining the strength and direction of serial effects in trustworthiness impressions. First, our findings on the influence of preceding stimulus exposure duration on the direction of serial effects align with prior research in face perception that separately reported attractive and repulsive biases depending on exposure, with longer durations determining negative aftereffects duration [29, 34, 39, 41, 43, 48, 81, 82, 88] and shorter leading to serial dependence [55, 56, 59, 64, 65, 67, 89]. Importantly, here we found for the first time serial dependence occurring for trustworthiness impressions, in contrast with a previous study which found no evidence [65]. This discrepancy likely arises from differences in stimulus selection. While Yu and Ying [65] used face images not specifically controlled for their level of trustworthiness, we employed faces systematically morphed along a trustworthiness continuum. This high level of control might have allowed us to isolate the trustworthiness dimension providing a more sensitive measure of trustworthiness serial effects.

This serial effect is unlikely to reflect only a response bias (response repetition), as it decreased with increasing relative distance, in accordance with the defining characteristics of serial dependence [31] (see “Experiment 2A and 2B”). In general, exposure duration to previous stimuli might be seen as a cue that the visual system uses to determine whether past and present visual information should be integrated or differentiated based on visual environment statistical temporal properties [31]. In fact, when a stimulus is viewed for a long time, any subsequent changes are likely to reflect changes in the actual visual environment. By contrast, brief exposure to a stimulus suggests that changes in the visual input are more likely to be due to noise in the visual input than actual changes in the visual environment. Consequently, negative aftereffects emerge after sufficiently long exposure durations.

Second, our findings show that identity similarity between adaptor and test face influences the direction of serial effects. When the previous and current faces shared a similar identity, we found an attractive bias towards the trustworthiness level of the previously seen face. In contrast, when the adaptor and test faces were selected from different morph identity continua, a repulsive bias emerged, pushing current trustworthiness judgements away from the previously seen face. These results align with previous research identifying similarity between stimuli as a key factor involved in modulating the strength and direction of serial effects [29, 59, 66, 83, 90, 91]. In the pivotal paper by Fischer and Whitney (2014), subsequent stimuli which differed more than 60° in orientation did not show a serial dependence effect. Similarly, negative aftereffect was found for larger relative stimulus differences [46]. The more two objects share similar visual features, the stronger the serial dependence bias manifests [29, 54, 67]. In serial dependence, feature similarity plays a crucial role in promoting perceptual stability [29, 55, 66]. When two consecutive stimuli are similar, smaller differences are less likely to be due to meaningful stimuli variations, but they instead might be due to noise in our visual input. Therefore, in such situations, our visual system tends to merge past and present visual information [31]. On the other hand, when two successive stimuli are very dissimilar, these differences are more likely to be due to actual object differences. Thus, our visual system tends to differentiate two stimuli as separate. As a result, serial dependence occurs when the feature difference between stimuli is small, whereas negative aftereffects are more pronounced when the adaptor and test stimuli are distinctly different. Interestingly, our results on feature similarity can be interpreted in light of the recently proposed Demixing model [92]. This model proposes that the visual system attempts to “demix” neural signals from stimuli presented close in time and space (e.g. the previous and current face) to accurately estimate their properties and identify whether they originated from the same or different sources. This model predicts that the direction and strength of serial biases are influenced by the amount of sensory noise and the discriminability of stimuli along the identifying dimension. In this model, highly discriminable stimuli, such as faces with different identities in our experiment, are readily demixed and thus induce repulsive biases. In contrast, low discriminable stimuli, such as subsequent faces that share the same identity, have reduced discriminability, eventually leading to a stronger attractive bias [92].

It is important to mention that, to measure the serial effects, our analysis considered morph similarity in trustworthiness ratings. Hence, we considered the perceived morph similarity, rather than the objective trustworthiness distance along the morph continuum. We determined the trustworthiness similarity between adaptor and test face by calculating the difference in morph units between the estimated scores (calculated from participants’ ratings) of the adaptor and test faces. While this approach allowed us to consider perceiver individual differences in trustworthiness judgements [4], it did not allow to measure objective morph trustworthiness differences between consecutive trials. Therefore, our study does not draw conclusions about differences in trustworthiness feature tuning between attractive and repulsive serial effects in trustworthiness impressions.

A third factor influencing the strength of serial dependence in trustworthiness impressions in our study was the spatial proximity between previous and current faces. In Experiment 2A, serial dependence strength was more pronounced when stimuli were presented sequentially in the same location, with the effect diminishing as the spatial distance between faces increased. These results reinforce the idea that serial dependence in trustworthiness impressions is spatially tuned, meaning it weakens with greater spatial distance between consecutive stimuli [29–31]. Importantly, the presence of spatial tuning shows that serial dependence in our experiments cannot be considered as a simple response bias, as it highly depends on the relative spatial location across faces. In our study, we considered serial dependence spatial tuning in world-centred coordinates (as participants could foveate towards the faces displayed on the screen), keeping the same retinotopic coordinates. Spatial tuning in serial dependence has been shown to occur both in world-centred coordinates, where participants fixate the stimuli appearing in different locations of the visual field [93, 94], and in retinotopic coordinates [29, 95]. Future studies could more directly examine the spatial tuning of trustworthiness serial effects by explicitly manipulating retinal coordinates, to provide further insights into the retinotopic or spatial invariant organisation of such serial effects for trustworthiness impressions in order to understand the level of visual processing at which serial dependence in trustworthiness impressions emerges.

Regarding negative aftereffects, in Experiment 2B we did not observe a reduction in the strength of face negative aftereffects based on the increasing relative spatial distance between the adaptor and test face. This result is not surprising, as participants were allowed to foveate to the face stimuli, therefore stimuli were consistently observed in the same retinotopic position (fovea) on each trial. Previous studies on spatial tuning for negative aftereffects have shown a decrease in the strength of face negative aftereffects with increasing spatial distance in retinotopic coordinates [85, 86]. While some studies found that face aftereffects are not retinotopic [34, 87], both low-level retinotopic processing and high-level face-selective mechanisms might contribute to face adaptation [36, 86]. Given that face negative aftereffects might have a retinotopic component [85], the lack of variation in negative aftereffects strength between spatial distance conditions in our study could be attributed to participants consistently foveating to the face stimuli, which maintained a constant retinotopic image position for the displayed faces across trials. In Experiment 2B we found a general negative aftereffect pattern for all the relative distance conditions, suggesting a robust negative serial bias.

Importantly, the strength of these biases was influenced by the exposure duration of the adaptor face, identity similarity and spatial distance between adaptor and test face. Our exploratory analysis (see: Interaction Between Time, Identity Similarity and Space) of the interactions between these factors revealed that while exposure duration shifts the global baseline of the serial bias toward either attraction or repulsion, the influence of identity similarity is strongest at closer spatial distances between successive faces and diminishes as spatial distance increases. These results suggest that both positive and negative serial effects can coexist simultaneously, and the combination of multiple factors determines their direction and strength. In this view, serial dependence and negative aftereffects can be additive effects [76, 83, 89], and it might be possible that both simultaneously occur, even if in certain conditions one is not strong enough to manifest clearly. Recent studies have shown that attractive and repulsive biases may occur simultaneously [46, 71, 73, 91, 92, 96–101]. Prior work demonstrated that our perception of orientation is attracted to very recent stimuli, then repelled by stimuli from the short to medium past, only to be re-attracted to those from the more distant past [100]. Repulsive biases have been shown to be more prominent when previous stimuli are ignored or irrelevant [71, 90, 91], when subsequent stimuli are very different in features [29, 68, 71], or when visual stimuli are high-contrast [101]. For example, Gekas et al. [101] found that stimulus contrast plays a crucial role in determining the direction of these serial effects, with high-contrast stimuli leading to a repulsive serial effect, while low-contrast stimuli leading to attractive serial effect. Taken together, these findings highlight that serial dependence is not a unitary phenomenon but rather the outcome of interacting factors that jointly shape whether perception is biased in an attractive or repulsive direction.

## Underlying mechanisms of serial dependence in trustworthiness impressions

Our results offer important insights into the debate on perceptual/post-perceptual mechanisms on serial dependence. It has been proposed that serial dependence occurs on the level of perception [28, 59, 66–68, 102, 103] decision [32, 70–74, 104] or memory [75–78] respectively. While there is no agreed definition on what these levels entail (potentially invalidating the debate to begin with), our results show that relatively simple perceptual decisions (such as trustworthiness ratings or medical judgements [105]) are heavily shaped by three main important stimulus properties: time, similarity and spatial location. Thus, any high-level account of serial dependence (decision and memory) would need to assume underlying processing of these basic factors on a trial-by-trial basis. It might be argued that decisions/memories are implicitly made about time duration, relative similarity, and spatial location of any stimulus, but this would blur any definition of decision/memory itself to encompass a wide range of features and factors. Independently on the level of processing, basic stimulus properties were shown to play a role in serial dependence [31], as well as attention [29, 83, 90], decision [32, 70–74, 104], confidence [106], and memory [75–78]. Thus, it is likely that all these levels continuously interact with each other. Still, it is important to highlight that there is strong evidence that serial dependence occurs in action, perception, decision and memory, independently on the locus/level of processing.

Regardless of the ongoing debate, our findings align with the idea that Continuity Fields [29, 31] play a continuous role in shaping visual experience. Continuity fields are spatially and temporally tuned mechanisms that, through serial dependence, smooth perception, actions, decisions, and memory over time. Their computational purpose is to enhance the accuracy, efficiency, and speed of information processing and perception, while supporting the stability of neural representations without requiring constant re-analysis of the entire visual scene [31]. This may be particularly relevant for initial judgements of trustworthiness—an important and frequent aspect of social perception. Notably, the temporal, spatial, and feature-based tunings we observe are consistent with the defining Continuity Fields properties [31].

It might be argued that our results are due to anchoring [107], a cognitive bias where judgements are influenced by a prior reference point (in this case the previous trust report), rather than perceptual serial effects. According to this view, in same identity trials, anchoring could lead to a bias toward the previous trustworthiness level seen as a reference, particularly when the two faces are similar in trustworthiness. In different identity trials, observers might show an “anti-anchoring” effect, when the current face differs substantially from the previous one. However, anchoring effects usually persist across multiple trials, as the first stimulus in a sequence is a reference for the following ones [107], whereas serial dependence is typically a short-term effect which decays quickly [29, 69], and thus our data are consistent with this pattern. While it is still possible that anchoring weakens over time (for example due to attentional drift from the adaptor face), our results show that the repulsive bias is stronger at longer adaptor duration (9 s), which is a result inconsistent with the idea of anchoring (or anti-anchoring) decaying with time. Instead, this result might suggest that prolonged exposure enhances visual adaptation, resulting in stronger repulsion in observers’ responses. Nevertheless, we recognise that manipulating spatial distance, duration, and stimuli similarity might also impact visual short-term memory encoding and thus do not unambiguously implicate perception. Therefore, together with the factors we manipulated, also memory or cognitive decision-making could plausibly contribute to the observed effects. Future research could more directly disentangle perceptual from post-perceptual influences by manipulating trial structure or memory load. Overall, our findings suggest that multiple factors interact to determine the strength and direction of serial effects biases in facial trustworthiness impressions, including the perceptual characteristics of previously seen stimuli related to exposure duration, similarity and spatial proximity. An interesting future direction would be to examine how these factors interact, as they are likely to operate in combination under everyday conditions.

Importantly, understanding any mechanisms that might affect trustworthiness impressions is informative not only theoretically, but has implications also on a practical level. Even though these impressions do not accurately reflect others’ personality traits or reliably guide our social decisions [6, 108, 109], they significantly influence our daily social interactions with deep consequences in society [14]. Given that we often encounter faces in succession rather than in isolation, it is plausible that biases from previously seen faces impact trustworthiness judgements in many real-world scenarios. For example, serial effects could hinder eyewitness identification during sequential police line-ups, where observers view photographs of potential suspects one after the other [110]. Understanding what causes attractive or repulsive biases towards previously seen faces is crucial in such circumstances. Prior work in criminal justice has already shown that sequential line-ups impair discriminability and memory compared to simultaneous line-ups, where the line-up members are displayed simultaneously [111]. Interestingly, a potential advantage of simultaneous line-ups might be the reduction of serial biases such as serial dependence and negative aftereffects introduced by viewing faces in a sequence. Similarly, in contexts like dating apps, where users browse through a series of profile pictures, the influence of previously viewed profiles on the trustworthiness impressions of current ones could have significant implications for romantic decisions [64].

## Trustworthiness impressions in the spatiotemporal context

In this study we focused on the influence of the temporal context on trustworthiness impressions. However, facial trait impressions are shaped not only by the temporal, but also the spatial context in which we perceive faces [5, 112, 113]. Every day we encounter faces not only in series but also in groups embedded in a spatial context. Previous research in the spatial domain has shown that the visual system can rapidly extract the average trustworthiness of a group of faces without the need to analyze each individual face in the crowd—a phenomenon known as ensemble perception [5, 114, 115]. Interestingly, this average group impression can then affect the judgements of trustworthiness of individual members of the group [116]. Therefore, trustworthiness impressions are malleable social judgements shaped by the spatiotemporal context in which faces are embedded. Overall, characterising under which conditions contextual biases in trustworthiness impressions arise is essential (see also [117]). Theoretically, it is particularly important to determine whether the principles underlying contextual biases for basic visual features also extend to such complex and high-level face social judgements. Practically, any intervention to reduce the influence of past visual experience on trustworthiness impressions would differ depending on the conditions that determine such biases. For example, with regard to serial dependence, its spatial and temporal tuning properties might be exploited to design face perception procedures, such as line-ups, that limit attractive visual biases.

While the results presented here further our understanding of the factors involved in determining the direction of serial effects in trustworthiness impressions, they also have some limitations. One limitation in the present study is the use of computer-generated FaceGen faces. These stimuli have some disadvantages, including lack of realism and differences in neural responses compared to natural faces [118, 119]. Nevertheless, we purposely chose these stimuli to enhance confidence that the observed serial effects reflected trustworthiness impressions. In fact, the FaceGen stimuli have been designed to vary mainly in trustworthiness appearance while minimising other potential confounds [120], allowing to control for confounding factors like gender and emotional expressions. In general, isolating trustworthiness while holding other facial features constant is challenging due to the complexity and intercorrelation of facial impressions with other social judgements [121–125].

## Conclusions

Taken together, our results show that trustworthiness impressions are not stable over time but are shaped by past visual experience both in a repulsive and attractive way. Our study characterised under which stimulus conditions negative aftereffects or serial dependence biases arise in trustworthiness impressions. Here, we were able to show a dissociation between these two serial effects by manipulating previous face exposure duration, identity similarity and spatial proximity between previous and current faces. Overall, these findings highlight the dynamic nature of facial trustworthiness judgements and provide insights into the mechanisms influencing serial effects in trustworthiness impressions, suggesting that decisional biases or memory alone cannot explain the observed modulation in direction (repulsive/attractive) and strength of the serial effects. Instead, previous face duration, identity similarity and spatial proximity are additional factors that should be considered in determining the strength and direction of serial effects in trait impressions.

## General methods

### Experiment 1A and 1B

#### Participants

In Experiment 1A (1 s adaptor condition), a total of 45 participants were recruited at the School of Psychology of the University of Aberdeen (UK) through SONA system (http://www.sona-systems.com/), word-of-mouth, and social media advertisement. Participants received either SONA credits or payment equivalent to £8/hour. This research was approved by the Ethics Committee of the School of Psychology of the University of Aberdeen (UK) and conformed to the Declaration of Helsinki. All participants gave informed consent. As exclusion criteria, first we excluded from the analysis participants with an error rate greater than 30% on the Asterisk attention control task (see “Procedure” description), indicating that they were not paying attention to the face stimuli displayed on the screen (ten participants). Second, for each participant we calculated the correlation between their responses on the trustworthiness rating task and the target faces displayed, and we removed from the analysis participants with a r-value less than 0.2, as it indicates random responses (four participants). Third, we removed from the analysis participants that did not use all the possible ratings across the whole experiment at least once (six participants). Fourth, after completing the experiment, participants completed a questionnaire to report any issues during the completion of the experiment, such as distraction, misunderstanding of the instructions or technical problems. Participants who indicated for any of the mentioned reasons that their data should be excluded were eventually removed from the analysis (zero participants). The final sample for Experiment 1A included 25 participants (21 females, 4 males; M = 22.7 years, SD = 6 years).

In Experiment 1B (9 s adaptor), 45 different participants were recruited using the same process and exclusion criteria as Experiment 1A. Nine participants were excluded from the analysis as their error rate was greater than 30% on the Asterisk attention control task, suggesting low attention during the completion of the experiment. Six participants were excluded as their correlation between rating scale responses and face morphs displayed on the screen had an *r*-value less than 0.2, indicating random responses. Third, we removed from the analysis participants that did not use all the possible ratings across the whole experiments at least once (five participants). The final sample for Experiment 1B included 25 participants (20 females, 5 males; M = 22.6 years, SD = 4 years). All participants provided informed consent prior to participation.

Several factors were considered to determine 25 participants as final sample size. We conducted a power analysis using G*Power and found that a sample size of 15 participants would be sufficient to achieve a power of 0.80 at α = 0.05 in a serial dependence experiment on face perception. This calculation was informed by a meta-analysis conducted by Manassi et al. (2023) on serial dependence, which calculated the effect sizes related to serial dependence for other attributes of face perception known to influence the perception of face trustworthiness, in particular face attractiveness and emotional expressions [3]. We calculated the average of the two effect sizes (serial dependence in emotion Fisher Zr = 0.6; face attractiveness Fisher Zr = 0.2), obtaining Fisher Zr = 0.4 (d = 0.8) as the effect size considered to run this power analysis. Additionally, since the goal of the present study was to investigate also negative aftereffects, we also ran a power analysis based on our recent study [43] (*d* = 0.68) which suggested that 19 participants would be sufficient to achieve the desired power level. To remain conservative and ensure robust statistical power in both experiments, we decided to use a sample size of 25 participants for both Experiment 1A and 1B, a decision also guided by sample sizes in related prior research [62, 64, 65].

#### Apparatus and stimuli

PsychoPy software (https://psychopy.org/) [126] was used to design and run the experiment. Stimuli were shown on a Sony TRINITRON CPD-G500 monitor, with participants positioning their heads in a chin rest placed at 50 cm away from the screen. The stimuli we used were computer-generated face images from FaceGen database [120], designed to vary in the level of perceived facial trustworthiness. From this database we chose three face identities, each with two corresponding versions, one trustworthy-looking and one untrustworthy-looking. Since prior work has shown that gender can influence perceived facial trustworthiness [127], with female faces generally being seen as more trustworthy than male-looking faces, we selected only male-looking identities to prevent gender from being a confounding factor. For the same reason, we decided to set the untrustworthy-looking morphed faces at −3 standard deviations on the trustworthiness dimension, and the trustworthy-looking versions only at + 1 standard deviation. In this way, we avoided trustworthy-looking faces to appear increasingly feminine with highest levels of trustworthiness [5, 14, 128]. To control for low-level differences across images, the images were converted in grayscale by using SHINE toolbox [129] in MATLAB R2017b (The MathWorks, USA), and their luminance and contrast were adjusted to align with the average values of the database. Importantly, here we employed faces controlled to vary specifically in their level of trustworthiness, differently from Yu and Ying (2021) that used faces not specifically selected or controlled for their level of trustworthiness. Therefore, we were able to precisely control similarity between sequential stimuli on the relevant perceptual trait participants were asked to rate. For each identity, we generated a face morph continuum of 51 images, ranging from the least untrustworthy-looking face (morph 0) to the most trustworthy-looking face (morph 50). The morphing procedure was conducted by using PsychoMorph software [130]. Face images were displayed at a visual angle of approximately 10.2° in height and 7.8° in width and were presented on a grey background.

#### Procedure

On each trial, a randomly selected adaptor face from the morph continuum of one of the three identities was displayed for 1 s (Experiment 1A) or 9 s (Experiment 1B). Participants were then instructed to rate the perceived level of trustworthiness of this face on a rating scale from 1 (not at all trustworthy) to 7 (extremely trustworthy) by using the keyboard. After 500 ms of inter-stimulus-interval, another face randomly selected from the morph continuum of one of the three identities was shown for 1 s (test face), and participants were asked to rate its level of trustworthiness on the same scale. The individual identities displayed for both the adaptor and test faces were randomly chosen, thus they were not necessarily the same identities within each trial. After a 500 ms inter-trial-interval (ITI), the next trial started (Fig. 1B). To check that participants were paying attention to the adaptor faces displayed on the screen, an attention control asterisk task was included. In 10% of the trials an asterisk quickly appeared on the top of the adaptor face for 150 ms and participants were asked to press spacebar as soon as they saw the asterisk. Observers with an error rate exceeding 30% on this task were excluded from analysis, as it suggests low attention during the completion of the experiment.

In Experiment 1A, participants completed six blocks of 56 trials (336 trials in total). Since the duration of the adaptor and test stimuli (1 s) and the intervals between them (ISI and ITI = 500 ms) were identical, there was no functional distinction between the adaptor and the test stimuli from the participant’s perspective. Consequently, in Experiment 1A, we analyzed serial biases in each trial relative to the previously seen stimulus regardless of the label of “adaptor” or “test” face. This approach allowed us to utilise the full dataset, and it aligns with the established serial dependence paradigm [28–32, 53]. However, in Experiment 1B, the adaptor and test faces differed in duration (9 s vs 1 s). Therefore, we increased the number of trials in Experiment 1B (672) compared to Experiment 1A (336 trials) to have the same number of trials considered in the analysis across experiments. In Experiment 1B, participants completed twelve blocks of 56 trials (672 trials in total: duration 9 s = 336 trials, duration 1 s = 336 trials). At the end of each block participants were encouraged to take breaks. Prior to starting the experiment, practice trials allowed participants to familiarise themselves with the stimuli and the task. First, participants completed 10 trials where a face randomly extracted from the face morph of one of the three identities was displayed 1 s, and they were instructed on how to rate the face. They then completed 10 practice trials with the asterisk task, which were identical to the actual experiment trials. Experiment 1A lasted approximately 30 min and Experiment 1B lasted 90 min. In the final part, participants were asked to type whether they encountered any issue during the completion of the experiment, such as distraction, misunderstanding of the instructions, or technical problems, and if consequently their data should be excluded from the analysis.

### Experiment 2A and 2B

#### Participants

Forty participants were recruited for Experiment 2A (1 s adaptor condition). The recruitment procedure and criteria for excluding participants were the same as Experiment 1A and 1B. Participants who showed an error rate over 30% in the Asterisk attention control task were excluded from the analysis (11 participants), indicating low attention during the experiment. Similarly, individuals whose correlation between rating scale responses and displayed face morphs had a r-value less than 0.2 were also excluded (2 participants), suggesting random responses. We also removed from the analysis participants who did not use all the possible ratings across the whole experiments at least once (2 participants). Additionally, participants experiencing issues such as distraction, technical difficulties, or misunderstanding of instructions (0 participants) were excluded from analysis. Twenty-five individuals (22 females, 3 males; M = 23.6 years, s.d. = 4 years) were ultimately selected for this experiment.

Thirty-seven participants were recruited for Experiment 2B (9 s adaptor condition). Following the exclusion criteria used in the previous experiments, we removed from the analysis participants whose error rate in the asterisk rating task was greater than 30% (7 participants), those whose correlation between rating scale responses and presented face morphs had a r-value less than 0.2 (4 participants). We also removed from the analysis participants who did not use all the possible ratings across the whole experiment at least once (1 participant), and participants who encountered issues during the experiment, such as distraction (0 participants), technical problems (0 participants), or misunderstanding of instructions (0 participants). Eventually, 25 participants (18 females, 6 males, 1 other; M = 28.7 years, s.d. = 11.5 years) were included in the analysis for this experiment. Participants gave informed consent before participating. The research was approved by the Ethics Committee of the School of Psychology of the University of Aberdeen (UK).

### Apparatus, stimuli, and procedure

Apparatus and stimuli were the same as in Experiment 1A and 1B. Differently from the previous experiments, on each trial both the adaptor and test faces were presented in random locations. Specifically, the *x* and *y* spatial coordinates of the face stimuli presented on the screen were randomly extracted from a range extending from 0 to ± 15 degrees of visual angle. This range was chosen because it allowed sufficient spatial variability to test for spatial tuning effects.

### Data analysis

Across the experiments, the following analyses were conducted:

#### Discriminability analysis

This analysis aimed to test whether observers were able to discriminate trustworthiness in the displayed face morphs. We correlated participant’s ratings (1–7) with the morphs (0–50 morph units) presented on each trial, so that for each participant we obtained a correlation value. We then determined if on a group level this correlation was significantly different from zero and between groups.

#### Serial effect analysis

This analysis aimed to identify and measure the strength and direction of any serial bias in the trustworthiness rating task. First, we calculated the trustworthiness judgement error for each participant on every trial. This error was computed as the difference between (1) participants’ trustworthiness ratings and (2) the expected rating (i.e. the “estimated score”) for the target face shown on that trial. To generate the “estimated scores,” we plotted each participant’s ratings as a function of the 51 possible target face morph trustworthiness levels. Second, we fitted a probit function to predict the rating values for each of the 51 face morph levels. This approach resulted in 51 estimated scores per participant, each ranging from 1 to 7. These 51 estimated scores represented participants’ typical perceived trustworthiness for each face morph level. Calculating the estimated scores allowed us to determine how much a participant’s rating on any given trial deviated from their own typical perception of trust of that specific face. Next, for each observer on each trial we calculated the difference in trustworthiness between the current and previous face stimuli by subtracting the estimated score of the current face morph from that of the previous. Trials with a response time exceeding 5 s were removed from the analysis.

In order to test whether a serial effect has occurred, we grouped together all participants’ judgement error and the difference in trustworthiness between the current and previous face stimuli. Next, we computed the running mean of the pooled trustworthiness judgement errors across all previous and current morph trustworthiness distances within a 2-morph-unit window. Due to its symmetrical nature around zero and to reduce noise, we simplified the data by flipping the left side of the running mean (negative morph distances) and merging it with the right side (positive morph distance), creating a “flipped running mean” made of only positive morph distances. Since there was a scarce number of trials for relative trustworthiness distances between the previous and current morph beyond 4, we limited our analysis to trials with relative distances ranging from 0 to 4 to avoid edge effects. Finally, we fitted a Derivative-of-Gaussian (DoG) to the flipped running mean. The DoG was calculated with the formula *y* = abcxe^−(bx)2^. Here, y represents the trustworthiness judgement error for each trial (participant’s rating − participant’s estimated score for the current target face); *a* is the half-amplitude of the DoG curve; *b* is the width of the DoG curve; c is a constant value √2/e^−0.5^ involved in making the *a* parameter equal to the half-amplitude of the curve peak: *x* represents the trustworthiness difference in estimated scores between the current and previous trial face morph. A positive value indicates an attractive bias (serial dependence) towards the previous face’s trustworthiness, while a negative value signifies a repulsive bias (negative aftereffect) away from it. A zero value means no serial bias.

#### Bootstrap analysis

At a group level, we ran 1000 iterations with replacement to compute a bootstrapped flipped moving average and fitted the DoG to measure serial effects direction and strength. Specifically, on each iteration, first participants from the sample were randomly sampled with replacement [131], and their trials were combined together, with a different mix of participants on each iteration. For each iteration, we calculated the flipped running mean of the trustworthiness judgement error as a function of the trustworthiness difference between adaptor and test face for the pooled data. Next, we fitted a DoG to the flipped running mean and calculated the half-amplitude of the curve. After 1000 iterations, we obtained a bootstrapped distribution of half-amplitudes and flipped running means. Eventually, we calculated the average of the flipped running means and generated the confidence intervals of the bootstrapped distribution. We also calculated the one-tailed *p*-values as the proportion of bootstrapped half-amplitude that was greater or equal to zero (bootstrap against zero). This analysis allowed us to measure the direction and strength of the serial effect. The unflipped, original bootstrapped moving averages are also reported in the Supplementary Materials (Additional file 1: Figs. S1, S2, S3). To compare different bootstrap distributions, we subtracted one half-amplitude distribution from another. We then calculated the one-tailed *p*-value by determining the proportion of values in the difference distribution that exceeded zero (Bootstrap within-subject for the same experimental group, Bootstrap between-subject for different experimental groups).

#### Similarity analysis

This analysis tested whether the serial effect direction and strength was modulated by the identity similarity between the current test face and the previously seen adaptor face. As in the Serial Effect Analysis, we calculated each participant’s trustworthiness judgement error and grouped all data. However, in this analysis, the “same identity trials” (i.e. trials in which the adaptor and test face morphs were extracted from same morph continuum identity) and “different identity trials” (i.e. trials in which the adaptor and test face morphs were extracted from different morph identity continuum) were analysed separately. The serial effect for each similarity condition was measured as described in the “Serial effect analysis” section. Given that the morph identity on each trial was randomly selected and we employed three morph identities, the “different identity” condition naturally had more trials. Therefore, in a subsequent bootstrap analysis, we ensured comparability by sampling with replacement the same number of trials for both “same” and “different identity” conditions.

#### Spatial tuning analysis

In Experiment 2A and 2B, we performed the same analyses as those conducted in Experiment 1A and 1B, with the only addition of the spatial tuning analysis. This analysis aimed to investigate whether the strength of serial effects for trustworthiness impressions is enhanced or weakened by spatial proximity between adaptor and test face. For each participant, we calculated the spatial distance in degrees between the position on the screen of the face morph in the previous and current trial. Next, we binned each participant trials in three different spatial distance ranges: trials with a spatial distance from 0 to 10 degrees of visual angle (short distance), trials with a spatial distance from 10 to 20 degrees of visual angle (medium distance), and trials with a spatial distance from beyond 20 degrees (long distance). Possibly due to fewer trials in this analysis, the moving average curves for the long-distance condition were often irregular, making the DoG fit unreliable during the bootstrap analysis. For this reason, in this analysis, instead of fitting a DoG, we calculated the mean area under the flipped moving averages for each spatial distance curve by calculating the average of all the *y*-axis values. Positive magnitude values (curve points above zero) indicate an attractive bias, while negative values (curve points below zero) indicate a repulsive bias. This method resulted in three distribution of 1000 “magnitude” values, one for each spatial distance condition.

## Supplementary Information

Supplementary Material 1: Figures S1–S3. Result graphs of Experiment 1A, 1B, 2A and 2B using the fullrunning mean. The x-axis shows trustworthiness judgement errors, the y-axis shows the relative trustworthiness distance in estimated scores between previous and current morph, and shaded areas represent confidence intervals. Fig. S1: Experiment 1 Results – Full Moving Average. Average of bootstrapped running meansand DoG fits for Experiment 1A and 1B. Fig. S2: Experiment 2 Results – Full Moving Average. Average bootstrapped running meansand DoG fits for Experiment 2A and 2B. Fig. S3: Experiment 2 Spatial Tuning Results – Full Moving Average. Average of bootstrapped running means not collapsed for Experiments 2A and 2B, shown across spatial distance bins between adaptor and test face. Fig. S4. Experiment 1B and 2B Test Face 9 s Duration Analysis. This graph contains the average of bootstrapped running means and DoG fits for Experiment 1B and 2B when considering the face morph shown for 9 s as “test faces” and the face morphs shown for 1 s as “adaptor faces”. The x-axis shows trustworthiness judgement errors, and the y-axis shows the relative trustworthiness difference in morph face trust between the previousand currentmorph. Shaded areas are confidence intervals

## Data Availability

All data generated or analysed during this study are included in this published article, its supplementary information files and publicly available repositories. The dataset supporting the conclusions of this article is available in the OSF repository https://osf.io/nyf5u/view_only=8029a04f584e44079bd3e70c892801b6.
