# Supplementary material for: Time, space and feature similarity determine attractive and repulsive serial biases in trustworthiness impressions
Source: BMC Biol. 2026 Jun 10;24:136. doi: 10.1186/s12915-026-02650-3 (PMC13251037; doi:10.1186/s12915-026-02650-3)
Supplement: Supplementary file 1 — Supplementary Material 1: Figures S1–S3. Result graphs of Experiment 1A, 1B, 2A and 2B using the fullrunning mean. The x-axis shows trustworthiness judgement errors, the y-axis shows the relative trustworthiness distance in estimated scores between previous and current morph, and shaded areas represent confidence intervals. Fig. S1: Experiment 1 Results – Full Moving Average. Average of bootstrapped running meansand DoG fits for Experiment 1A and 1B. Fig. S2: Experiment 2 Results – Full Moving Average. Average bootstrapped running meansand DoG fits for Experiment 2A and 2B. Fig. S3: Experiment 2 Spatial Tuning Results – Full Moving Average. Average of bootstrapped running means not collapsed for Experiments 2A and 2B, shown across spatial distance bins between adaptor and test face. Fig. S4. Experiment 1B and 2B Test Face 9 s Duration Analysis. This graph contains the average of bootstrapped running means and DoG fits for Experiment 1B and 2B when considering the face morph shown for 9 s as “test faces” and the face morphs shown for 1 s as “adaptor faces”. The x-axis shows trustworthiness judgement errors, and the y-axis shows the relative trustworthiness difference in morph face trust between the previousand currentmorph. Shaded areas are confidence intervals [file 12915_2026_2650_MOESM1_ESM.pdf]

Supplementary Materials

Bootstrap Running Mean Not Collapsed

This section presents the result graphs of Experiment 1A, 1B, 2A and 2B using the full (unflipped) running mean.

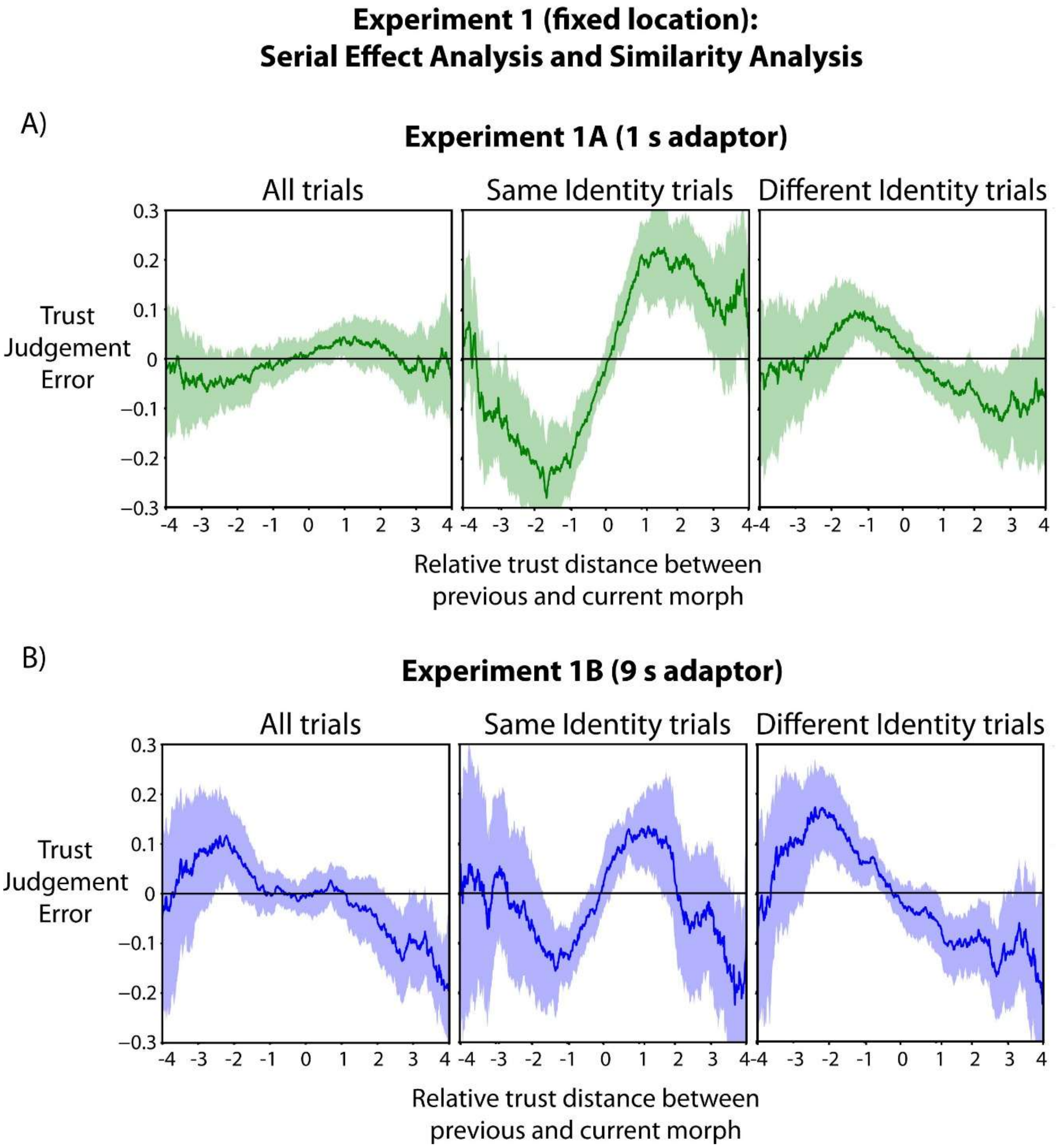

Supplementary Figure 1: Experiment 1 Results – Full Moving Average.

Average of bootstrapped running means (unflipped) and DoG fits for Experiment 1A (A; green) and 1B (B; blue). The x-axis shows trustworthiness judgement errors, and the y-axis shows the relative trustworthiness distance in estimated scores between previous and current morph. Shaded areas are confidence intervals. The similarity analysis is also reported for experiment 1A and 1B.

Experiment 2 (random location):
Serial Effect Analysis and Similarity Analysis

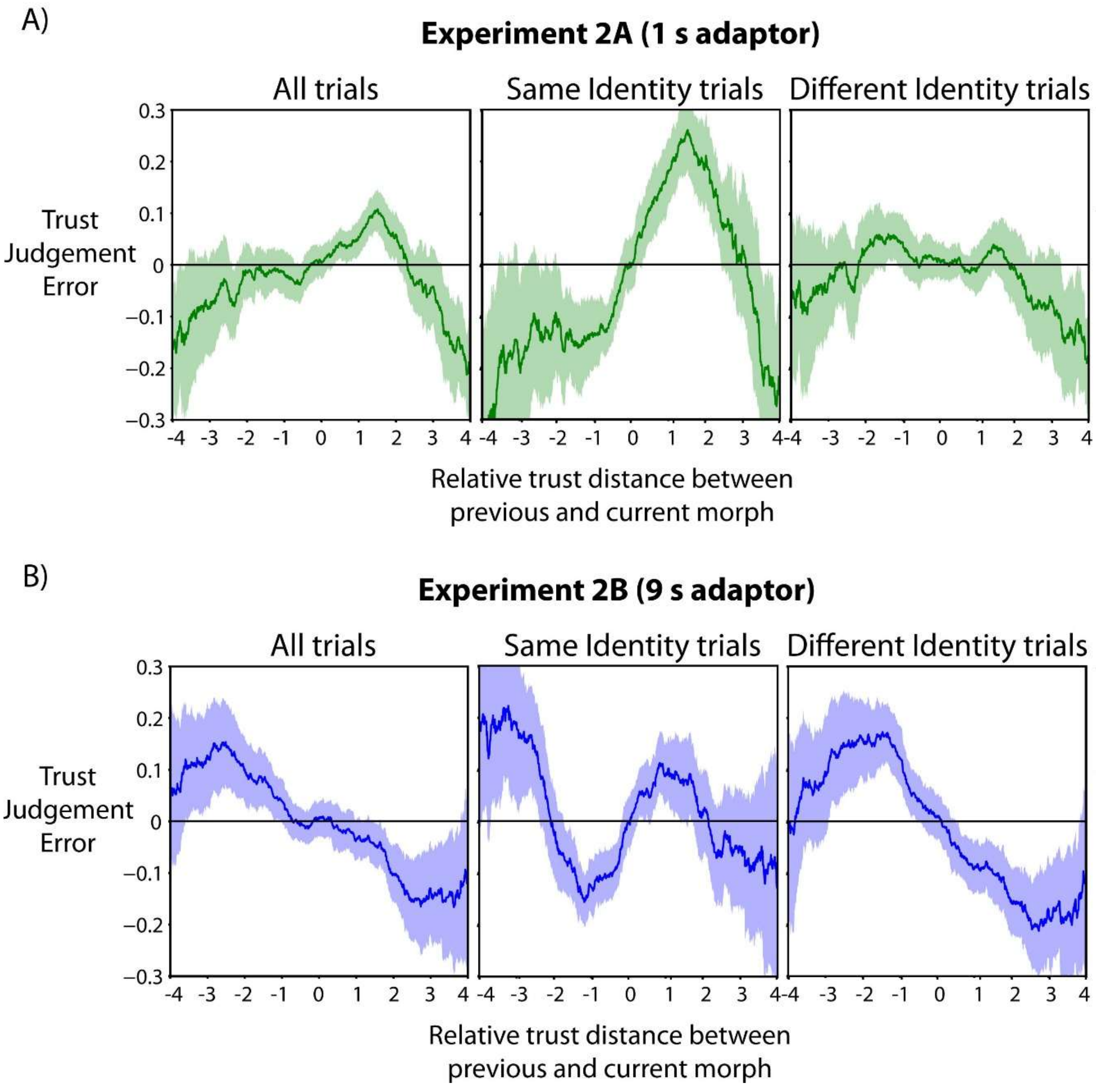

Supplementary Figure 2: Experiment 2 Results – Full Moving Average.

Average bootstrapped running means (unflipped) and DoG fits for Experiment 2A (A; green) and 2B (B; blue). The x-axis shows trustworthiness judgement errors; the y-axis shows the relative trustworthiness distance in estimated scores between previous and current morph. Shaded areas indicate confidence intervals. Similarity analyses for Experiments 2A and 2B are also included.

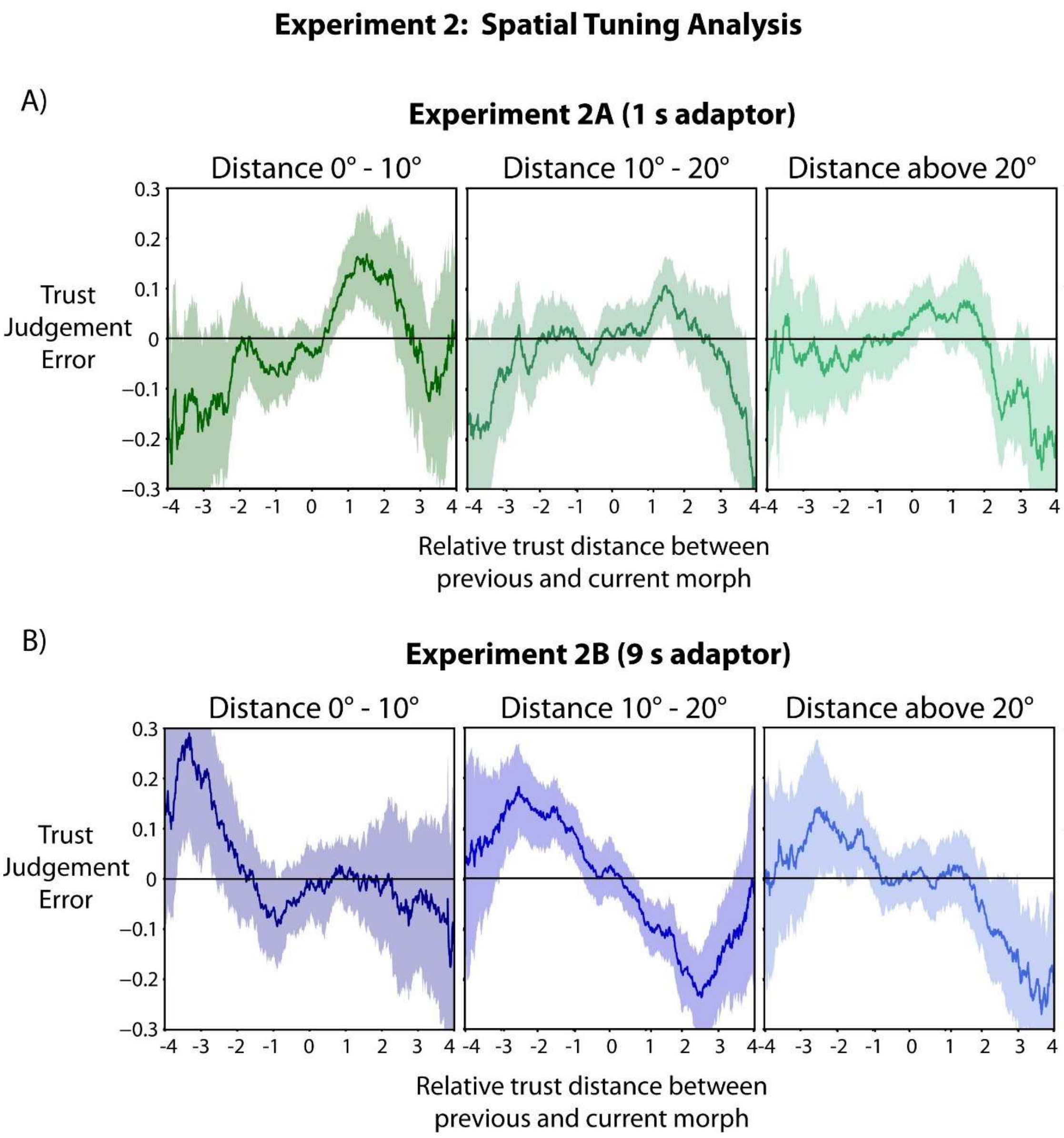

Supplementary Figure 3: Experiment 2 Spatial Tuning Results – Full Moving Average.

Average of bootstrapped running means not collapsed for Experiments 2A (A) and 2B (B), shown across spatial distance bins between adaptor and test face (0–10°, 10–20°, >20°). The x-axis shows trustworthiness judgement errors the y-axis shows the relative trustworthiness distance between previous and current face morph. Shaded areas indicate confidence intervals.

The Influence of Test Face Duration on Serial Effects

We ran an exploratory analysis to investigate whether the duration of the test stimulus, and not just the preceding adaptor's duration, is an additional factor in determining the strength and direction of serial effects in trustworthiness impressions. Since in Experiment 1B and 2B the face stimuli were presented for two different durations (adaptor face: 9 s; test face: 1) differently from

Experiment 1A and 1B (adaptor and test face: 1 s), we focused our analysis on these two experiments. Specifically, here we considered "test faces" the stimuli shown for 9 s, and "adaptor faces" the stimuli displayed for 1 s. We measured how participants' judgements of trustworthiness of the current 9 s face were biased towards or away from the previously seen 1 s face's level of trustworthiness. We employed the same Serial Effect analysis described in the main manuscript.

In Experiment 1B, we found a negative half-amplitude of -0.11 (bootstrap against zero; p-value < .001), indicating that trustworthiness judgements of the 9 s test faces were repelled away from the preceding 1s adaptor faces (i.e., negative aftereffects; Supplementary Figure 4). Similarly, in Experiment 2B, we found a negative half-amplitude of -0.09 (bootstrap against zero; p-value < .001), further demonstrating a bias in trustworthiness judgements on the 9 s test faces away from the 1 s adaptor faces (i.e., negative aftereffects; Figure 2B). Overall, our results showed negative aftereffects when the test face was presented for a longer duration (9 s) and preceded by briefly shown (1 s) face.

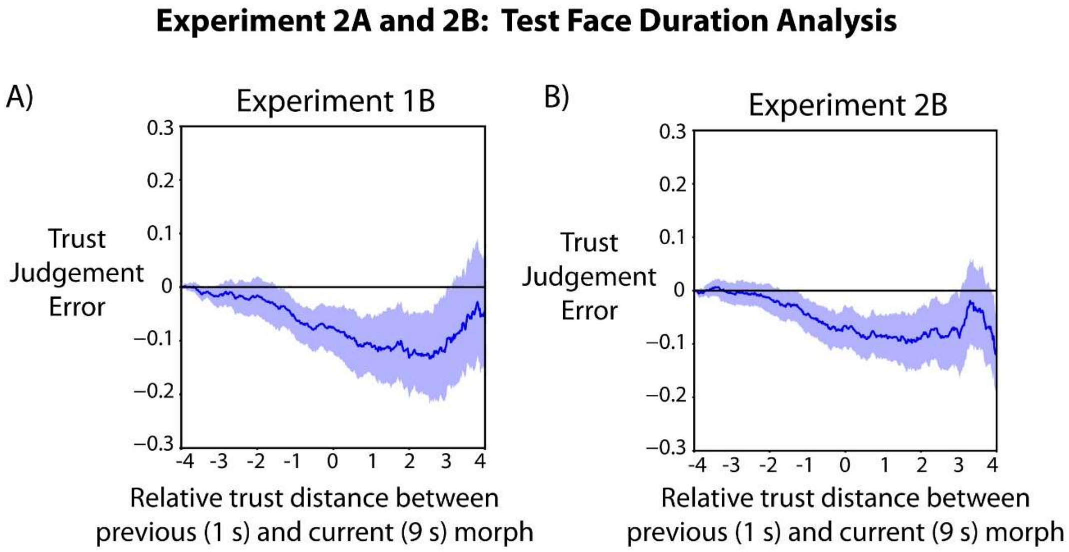

Supplementary Figure 4: Experiment 1B and 2B Test Face 9 s Duration Analysis.

Average of bootstrapped running means and DoG fits for Experiment 2B (A) and 2B (B) when considering the face morph shown for 9 s as "test faces" and the face morphs shown for 1 s as "adaptor faces". The x-axis shows trustworthiness judgement errors, and the y-axis shows the relative trustworthiness difference in morph face trust between the previous (1s) and current (9s) morph. Shaded areas are confidence intervals.

These results could be interpreted as due to the temporal tuning property of serial dependence, which implies that serial effect decays over time (Bliss et al., 2017; Fritsche et al., 2020: Fischer & Whitney, 2014; Manassi et al., 2023). It is possible that as time passes between the previous and current face stimulus, the attractive bias of serial dependence initially generated by the short duration face (adaptor 1 s) weakens and is eventually overtaken by a repulsive bias, in line with previous work by Bliss et al. (2017b). Indeed, if we consider the total time between the onset of the previous 1 s face and the trustworthiness judgement of the current 9 s face, the interval is approximately 10 seconds, which is a timeframe that has been associated with repulsion (Fritsche et al., 2020; Gekas et al., 2019b). Additionally, serial dependence is usually present when the current stimulus is brief, noisy, or ambiguous and highly uncertain (Cicchini et al., 2018; Gallagher & Benton, 2022). However, in the present analysis, the current stimulus was presented for 9 s, likely resulting in a highly certain representation. Therefore, the need for visual stabilisation through an attractive bias could have been minimised, allowing repulsion to manifest to better differentiate successive stimuli (Fritsche et al., 2020).
